# Identification of a novel glycolysis-related signature to predict the prognosis of patients with breast cancer

**DOI:** 10.1186/s12957-021-02409-w

**Published:** 2021-10-02

**Authors:** Menglin He, Cheng Hu, Jian Deng, Hui Ji, Weiqian Tian

**Affiliations:** Department of Anesthesiology, Affiliated Hospital of Nanjing University of Chinese Medicine, Jiangsu Province Hospital of Chinese Medicine, No. 155 Hanzhong Road, Qinhuai District, Nanjing, 210029 Jiangsu China

**Keywords:** Bioinformatics, Glycolysis, Gene signature, Breast cancer, Prognosis

## Abstract

**Background:**

Breast cancer (BC) has a high incidence and mortality rate in females. Its conventional clinical characteristics are far from accurate for the prediction of individual outcomes. Therefore, we aimed to develop a novel signature to predict the survival of patients with BC.

**Methods:**

We analyzed the data of a training cohort from the Cancer Genome Atlas (TCGA) database and a validation cohort from the Gene Expression Omnibus (GEO) database. After the applications of Gene Set Enrichment Analysis (GSEA) and Cox regression analyses, a glycolysis-related signature for predicting the survival of patients with BC was developed; the signature contained *AK3*, *CACNA1H*, *IL13RA1*, *NUP43*, *PGK1*, and *SDC1*. Furthermore, on the basis of expression levels of the six-gene signature, we constructed a risk score formula to classify the patients into high- and low-risk groups. The receiver operating characteristic (ROC) curve and the Kaplan-Meier curve were used to assess the predicted capacity of the model. Later, a nomogram was developed to predict the outcomes of patients with risk score and clinical features over a period of 1, 3, and 5 years. We further used Human Protein Atlas (HPA) database to validate the expressions of the six biomarkers in tumor and sample tissues, which were taken as control.

**Results:**

We constructed a six-gene signature to predict the outcomes of patients with BC. The patients in the high-risk group showed poor prognosis than those in the low-risk group. The area under the curve (AUC) values were 0.719 and 0.702, showing that the prediction performance of the signature is acceptable. Additionally, Cox regression analysis revealed that these biomarkers could independently predict the prognosis of BC patients with BC without being affected by clinical factors. The expression levels of all six biomarkers in BC tissues were higher than that in normal tissues; however, *AK3* was an exception.

**Conclusion:**

We developed a six-gene signature to predict the prognosis of patients with BC. Our signature has been proved to have the ability to make an accurate prediction and might be useful in expanding the hypothesis in clinical research.

## Introduction

In 2020, breast cancer (BC) was estimated to account for 30% of cancers in females, in which 15% of the cases lead to death [1, 2]. So far, the effects of novel biomarkers on diagnosis and prognosis are far from satisfactory [3]. Moreover, despite considering the option of lymph node dissection for the treatment of BC, there are still some limitations in clinical practice [4, 5]. Therefore, there is a need to construct an effective prognostic model for predicting the outcomes of patients with BC in clinical practice.

With the supply of adequate oxygen, normal cells metabolize glucose to pyruvate via glycolysis that is further used to produce adenosine triphosphate (ATP) through mitochondrial oxidative phosphorylation; whereas in the case of anaerobic or hypoxic conditions, normal cells ferment glucose to lactate [6]. However, even after a sufficient supply of oxygen, tumor cells mainly use the mechanism of glycolysis to produce energy and thus have a high glycolysis rate. This phenomenon is known as the Warburg effect, which is found in all types of cancer [7]. Many studies have proved that glycolysis can accelerate the proliferation, invasion, and migration of certain tumor cells and enhance drug resistance [8, 9]. Therefore, these glycolysis-related genes and proteins can be used as targets for prognosis or treatment in patients with BC. For instance, pieces of evidence have proved that key enzymes in the aerobic glycolytic pathway including hexokinase (HK), phosphofructokinase (PFK), and pyruvate kinase (PK) can serve as potential therapeutic targets for anti-tumor [10]. A previous study reported that the HKII, one isoenzyme of HK, is overexpressed in tumor cells [11]. PFK-1, a rate-limiting enzyme in glycolysis, promotes the conversion of fructose 6-phosphate to fructose 1,6-bisphosphate and adenosine diphosphate (ADP) [12]. This conversion is facilitated by 6-phosphofructo 2-kinase/fructose 2, 6-bisphosphatase (PFKFB). As a subtype of PFKFB, PFKFB3 has been found in patients with BC in high expression levels and was further implicated to a poor prognosis in clinical practice [13]. PKM2 is an isoenzyme of PK and is mainly located in muscles [14]. It has been reported that the overexpression of PKM2 is related to worse overall survival (OS) and progression-free survival (PFS) in patients with BC [14]. Among the 14 different types of glucose transporter protein (GLUT), the notable GLUT1 subtype was found to have a close correlation with the progression of tumor [15]. Li et al. found that the overexpression of GLUT1 is associated with more severe outcomes in patients with head and neck squamous cell carcinoma (HNSCC) that was further ascribed to the activation of the nuclear factor kappa B (NF-κB) signaling pathway [16]. Moreover, the expression level of GLUT1 increased significantly in the kidney cancer [17], ovarian cancer [18], and liver cancer [19].

With the development of bioinformatics, biomarkers are used in comparatively more comprehensive studies to predict the prognosis of patients [20, 21]. Nine glycolysis-related genes (GRGs) were identified as being closely relevant to worse outcomes in endometrial cancer [22] and glioblastoma [23], respectively. Another set of 11 GRGs prognostic model was constructed and served as an effective tool to predict prognosis and guide clinical practice [24]. Circ-FOXM1 has been demonstrated that it can contribute to cell proliferation and glycolysis in melanoma [25]. These studies made an effort to probe the effect of glycolysis, but the role of glycolysis-related genes involved in BC is far from understood. Therefore, it is of great importance to profoundly understand the effect of glycolysis on tumorigenesis and tumor progressions.

In this study, we conducted a six-GRGs risk signature to predict the prognosis of patients with BC by integrating high-throughput data. This prognostic model might become a part of clinical prognostic features and provides a novel insight into BC study.

## Materials and methods

### Data processing

**Fig. 1:**
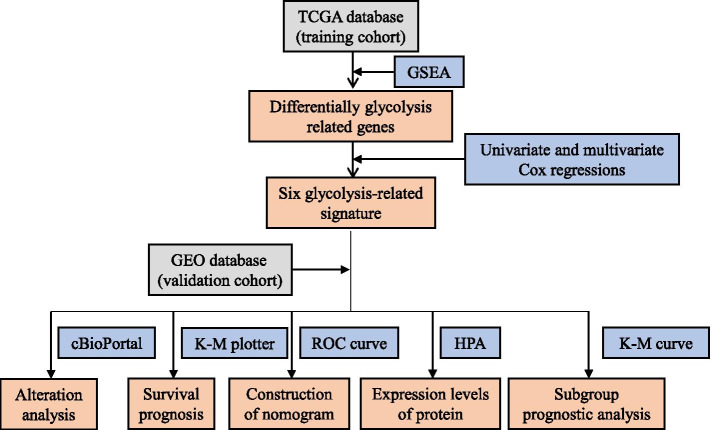
Flowchart of the experimental design

The overall design of this study is shown in Fig. 1. The gene expression and relevant clinicopathological data of patients with BC were downloaded from Genomic Data Commons (GDC) database (https://portal.gdc.cancer.gov/). A total of 1066 BC samples and 112 normal samples were included in our study, which was set as a training cohort. Whereas 198 BC samples in the *GSE25065* dataset were obtained from the Gene Expression Omnibus (GEO) of the National Center of Biotechnology Information (NCBI) database [26] and were set as a validation cohort, clinical information, including age, American Joint Committee on Cancer (AJCC) stage, grade, and TNM staging were presented (Table 1). Kaplan-Meier plotter (http://kmplot.com/analysis/) was performed to evaluate the prediction capacity of biomarkers.

**Table 1:** Clinical characters in patients with BC in TCGA dataset

Characters	Subgroup	Ratio
Age	≤ 60	581 (54.5%)
	> 60	465 (43.62%)
	Unknown	20 (1.87%)
AJCC stage	I	172 (16.35%)
	II	597 (56.75%)
	III	239 (22.72%)
	IV	20 (1.9%)
	Unknown	24 (2.28%)
T	I	270 (25.66%)
	II	614 (58.37%)
	III	129 (12.26%)
	IV	36 (3.42%)
	Unknown	3 (0.29%)
M	0	875 (83.17%)
	1	22 (2.09%)
	Unknown	155 (14.73%)
N	0	483 (45.91%)
	1	354 (33.65%)
	2	119 (11.31%)
	3	76 (7.22%)
	Unknown	20 (1.9%)
Status	Dead	138 (13.12%)
	Alive	914 (86.88%)

### Gene set enrichment analysis

Gene Set Enrichment Analysis (GSEA) (http://www.broadinstitute.org/gsea/index.jsp) was applied to determine whether there is any significant difference in the expression levels between BC tissues and normal tissues. Eight gene-sets related to glycolysis were downloaded from the Molecular Signature Database (https://www.gsea-msigdb.org/gsea/msigdb/index.jsp). Each gene set had 1000 permutations to get a normalized enrichment score (NES), false discovery rate (FDR), and normalized *P* value (*P*), wherein NES ≥ 1.5, FDR < 0.1, and *P* < 0.05 were set to screen the eligible gene set for the following analysis.

### Differential expression analysis and model construction of glycolysis-related genes

Firstly, we performed univariate Cox proportional hazard regression analysis to determine the relationship between glycolysis-related genes and OS. Genes with *P*<0.05 were deemed as candidate prognostic genes for BC. Then, multivariate Cox proportional hazard regression analysis was used to establish the prognostic model based on the abovementioned candidate prognostic genes. The risk score was calculated using the following formula: Risk score= ${\sum}_{\mathrm{i}=1}^{\mathrm{n}} coef\ast id$. The median value of the risk score was set as cut-off, and samples were divided into high- or low-risk groups. We conducted the Kaplan-Meier survival analysis to assess the difference in survival between the two groups using the R package survival. The time-dependent ROC curve was used to assess the accuracy of the model through the R package survival ROC. Similarly, the same signature also generates risk scores in each patient in the *GSE25065* dataset that could be used to validate the performance of the model.

### The glycolysis-related signature is an independent prognostic factor for breast cancer

Univariate, and multivariate Cox regression analysis, along with data stratification analysis was performed to assess whether the risk score was independent of the clinical characters, including age and grade. *P* < 0.05 was considered as statistically significant.

### cBioPortal analysis

The cBioportal for Cancer Genomic database (http://www.cbioportal.org) was used to analyze the forms and ratios of alterations in biomarkers in BC. All steps were performed complying with the instructions of the cBioPortal.

### Construction and validation of nomogram

The nomograms were developed to explore the prognosis of patients with BC in 1-, 3-, and 5-year overall survival. We further used calibration curves and concordance (C_index) to assess the predictive capacity of the nomogram, both in training and validation cohorts.

### Human Protein Atlas

The immunohistochemical (IHC) images in the Human Protein Atlas (HPA) database (https://www.proteinatlas.org/) were applied to validate the expression of biomarkers in tumor and normal tissues.

### Statistical analysis

R software and Perl languages (http://www.perl.org/) were used to perform all the statistical analyses. Before the analysis, the gene expression data were normalized via log2 transformation. The threshold of *P* < 0.05 was considered significantly different.

## Results

### Screening genes with gene set enrichment analysis

**Fig. 2:**
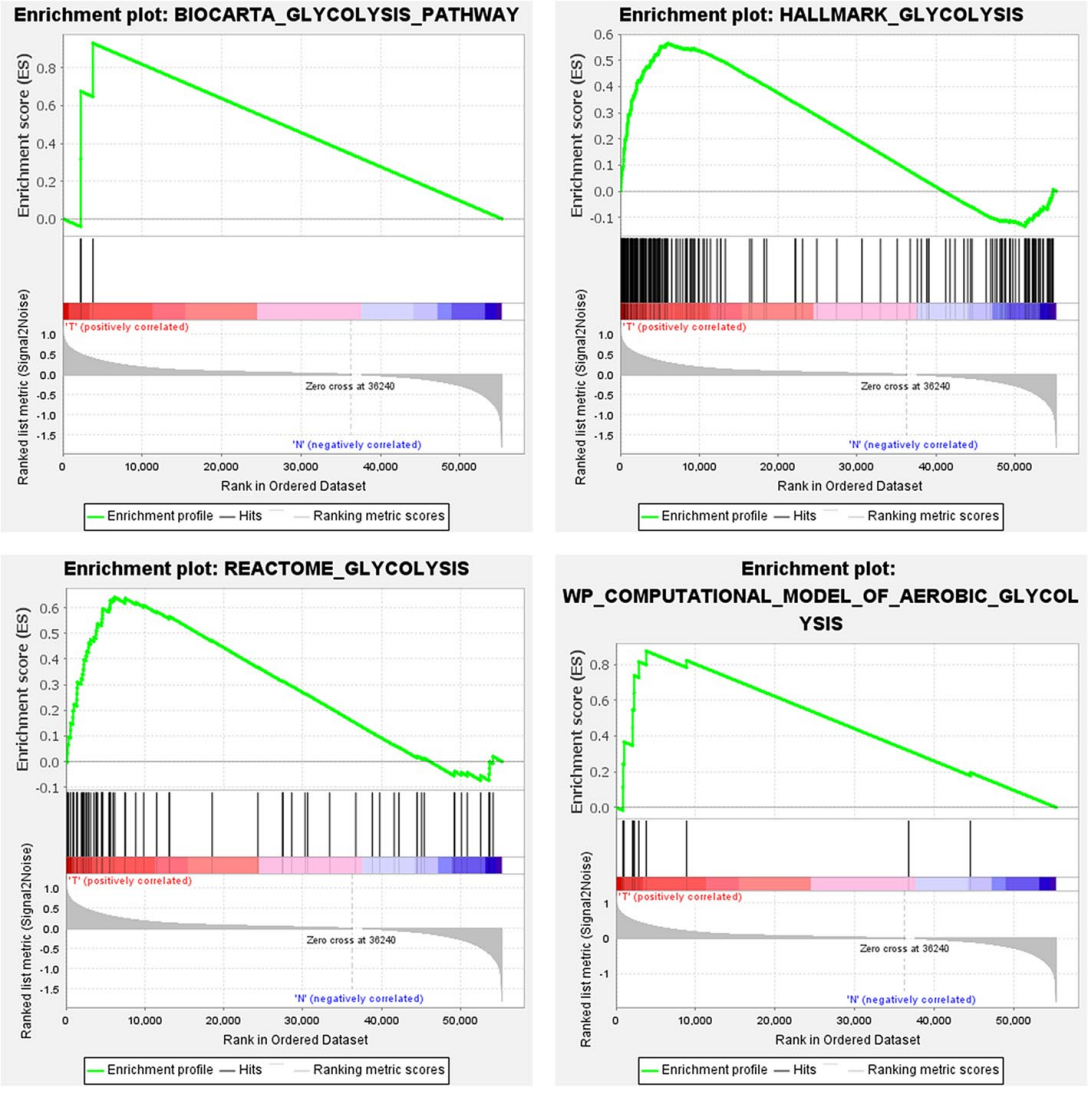
Enrichment of four glycolysis-related gene sets in GSEA. **A** BIOCARTA_GLYCOLYSIS_PATHWAY. **B** HALLMARK_GLYCOLYSIS. **C** REACTOME_GLYCOLYSIS. **D** WP_COMPUTATIONAL_MODEL_OF_AEROBIC_GLYCOLYSIS

We downloaded a dataset including the gene expression and clinical information on 1066 BC patients and 112 normal controls from THE Cancer Genome Atlas (TCGA). Eight glycolysis-related gene sets were downloaded, and a total of 410 genes were obtained. We used the data mentioned above and GSEA to analyze the possibility of significant differences in gene-sets between BC samples and normal samples and obtained four significantly enriched gene-sets in our study (Fig. 2). Next, we sorted out 284 glycolysis-related genes through the integration of these four gene-sets.

### Construction of a six-gene signature as a prognostic indicator

**Fig. 3:**
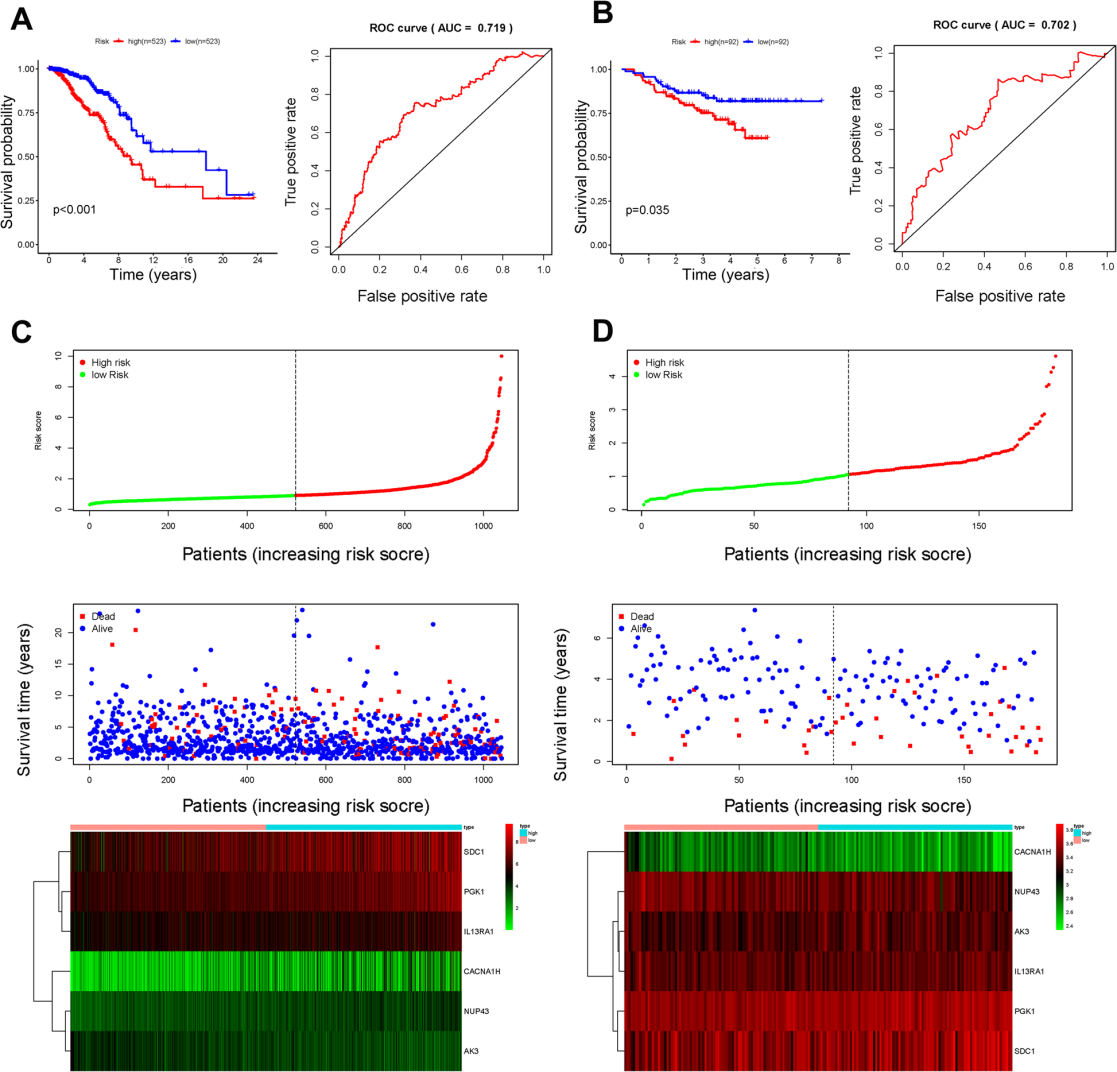
The prediction of our model on the prognosis of patients with BC. **A** Kaplan-Meier curve of survival and ROC curve in the training cohort. **B** Kaplan-Meier curve of survival and ROC curve in the validation cohort. **C** Risk score, survival status, and heat map of six biomarkers in training cohort. **D** Risk score, survival status, and heat map of six biomarkers in validation cohort

Univariate analysis was applied to analyze the 284 glycolysis-related genes, and eight genes (*AK3*, *CACNA1H*, *IL13RA1*, *NUP43*, *PGAM1*, *PGK1*, *P4HA2*, and *SDC1*) were obtained. Furthermore, multivariate analysis was performed to screen genes associated with the survival of patients with BC. Finally, six genes were identified as prognostic genes, with each gene having a coefficient value (Table 2). Risk score = coefficient gene 1 × expr (gene 1) + coefficient gene 2 × expr (gene 2) + … + coefficient gene n × expr (gene n), where expr is the expression of the corresponding gene. The median risk score was set as the threshold, and patients were divided into high- and low-risk groups. The Kaplan-Meier survival curve demonstrated that the survival rate of the high-risk group was significantly lower than that of the low-risk group (Fig. 3A). The area under the curve (AUC) value of the dependent ROC for the six-mRNA signature was 0.719, indicating that the signature could accurately predict the survival of patients with BC. The consistent results were obtained in the validation cohort (Fig. 3B). Moreover, the risk scores, survival status, and expression levels of biomarkers were drawn in both training and validation cohorts (Fig. 3C, D).

**Table 2:** Univariate and multivariate Cox regression analysis for glycolysis-related genes and respective coefficient

	Univariate Cox regression			Multivariate Cox regression		
Gene	HR (95% CI)	***P*** value	Gene	HR (95% CI)	***P*** value	Coefficient
AK3	0.964 (0.931‑0.997)	0.034	AK3	0.975 (0.942‑1.009)	0.145	−0.0255
CACNA1H	1.018 (1.005‑1.032)	0.008	CACNA1H	1.017 (1.004‑1.031)	0.013	0.01724
IL13RA1	1.009 (1.002‑1.016)	0.010	IL13RA1	1.005 (0.998‑1.013)	0.139	0.00545
NUP43	1.054 (1.022‑1.088)	***	NUP43	1.049 (1.016‑1.084)	0.003	0.04819
PGAM1	1.038 (1.006‑1.070)	0.019	PGK1	1.006 (1.003‑1.009)	***	0.00594
PGK1	1.008 (1.005‑1.010)	***	SDC1	1.002 (1.000‑1.004)	0.012	0.00215
P4HA2	1.069 (1.024‑1.116)	0.002				
SDC1	1.002 (1.001‑1.004)	0.002				

*HR* hazard ratio, *CI* confidence interval

***p < 0.001

### The prediction of biomarkers on prognosis was validated and used as an independent prognostic indicator in patients with breast cancer

**Fig. 4:**
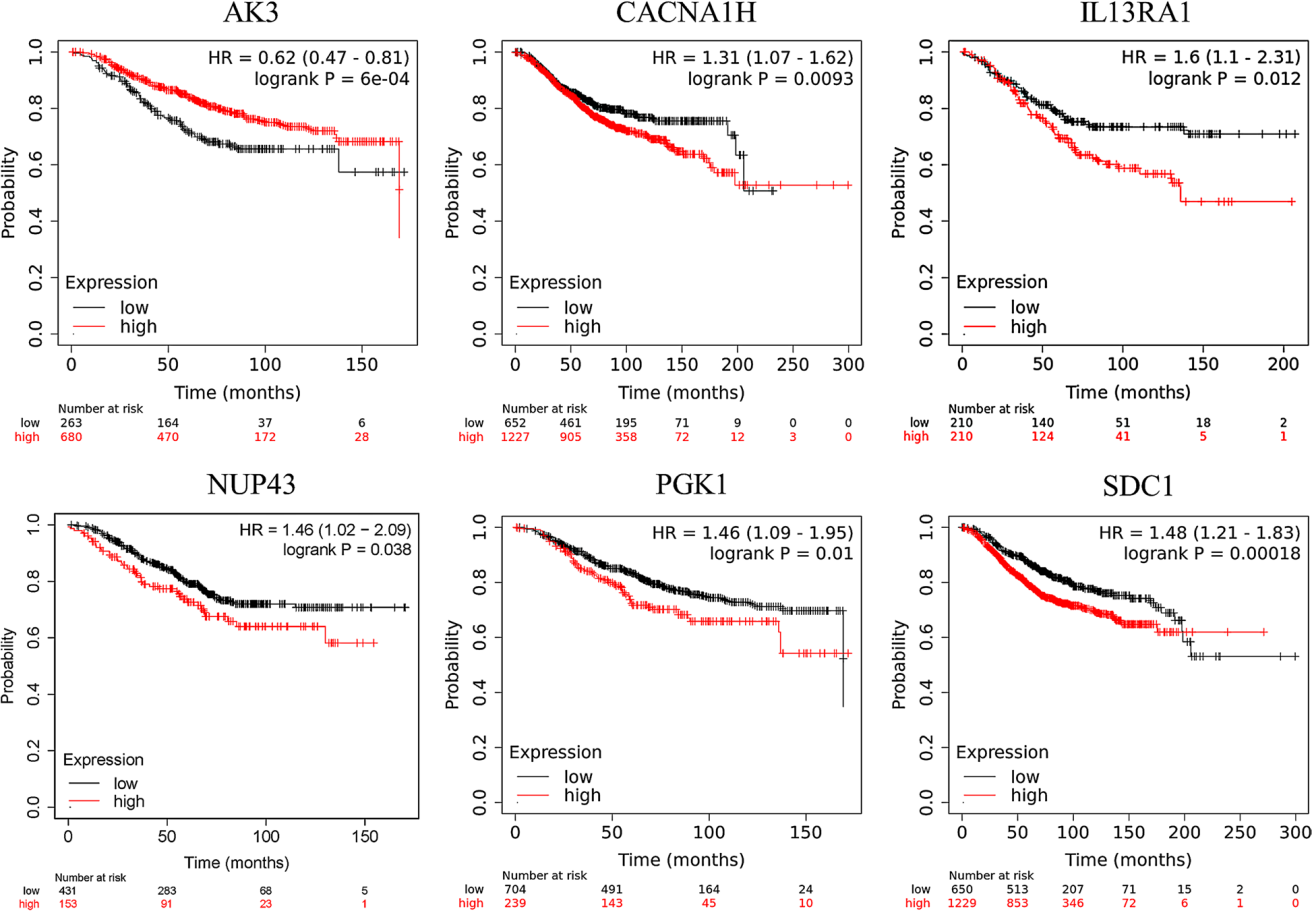
Forest plot of clinical characters and risk score. **A**, **B** Univariate and multivariate Cox regression analysis in training and validation cohorts

Kaplan-Meier plotter database was used to assess the prognostic ability of biomarkers. Patients were divided into two groups based on the expression levels of biomarkers, and the OS of patients were analyzed. We found that the high expression of the other five biomarkers is related to shorter OS, while that of AK3 indicates a better OS (Fig. 4).

**Fig. 5:**
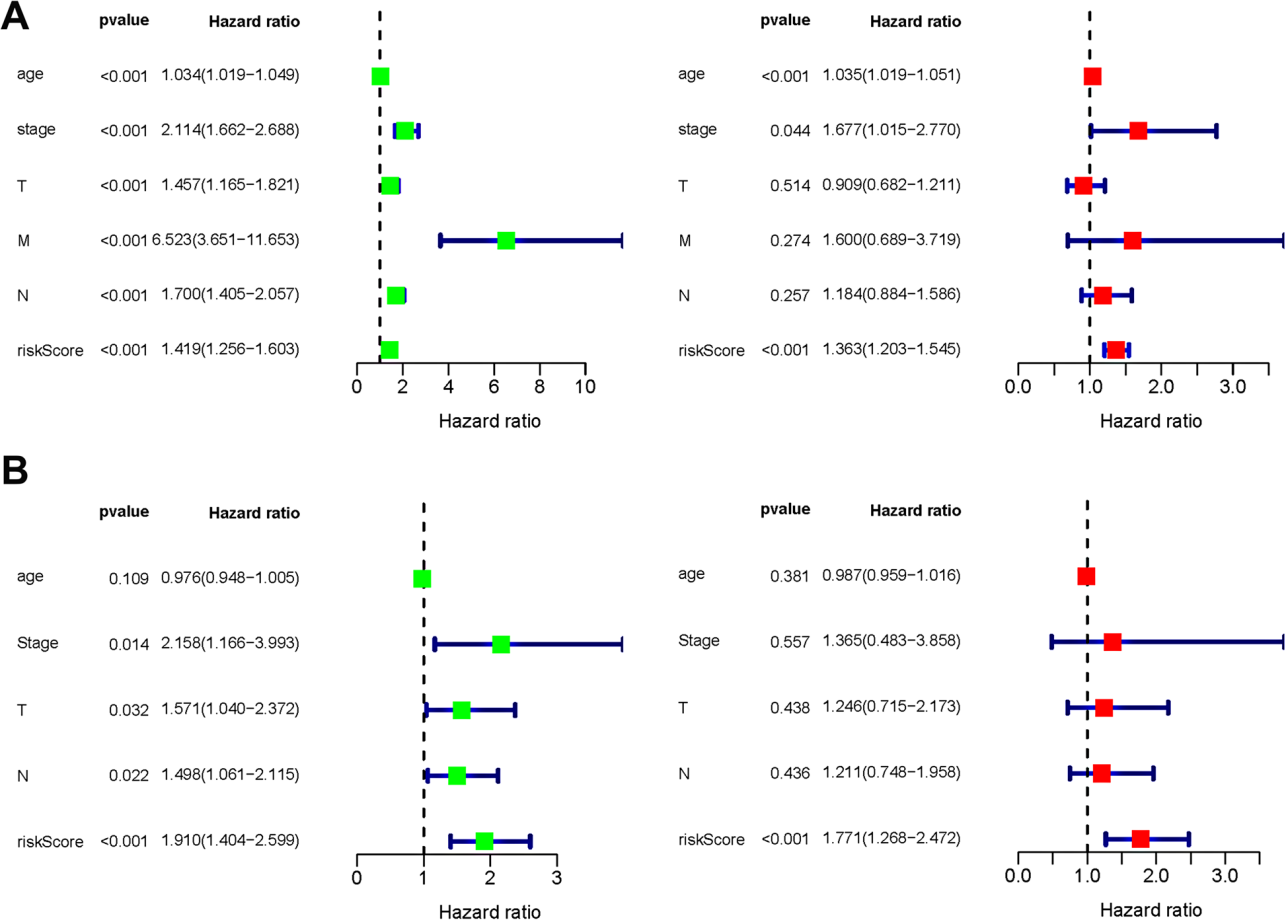
Overview of six biomarkers. **A** The alterations of six biomarkers in patients with BC. **B** The most common type of alterations in BC. **C** The expression levels of six biomarkers in normal and tumor samples

To assess the prognostic ability of biomarkers and to analyze whether they can be independent of clinical features, including age and grade, univariate and multivariate Cox regression analyses were performed. The results demonstrated that biomarkers could independently predict the survival of patients both in training and validation cohorts (Fig. 5A, B).

### Analysis of the biomarkers

**Fig. 6:**
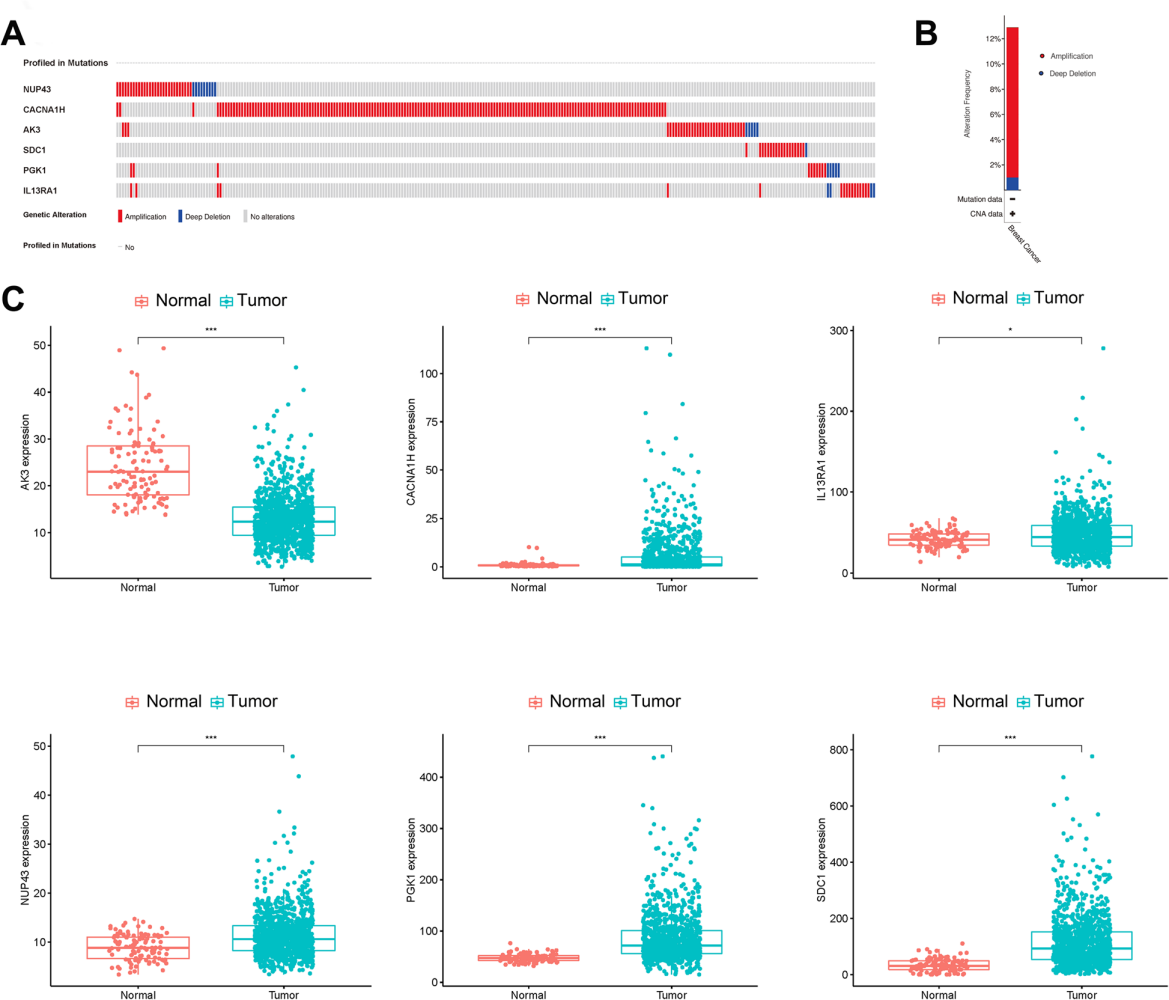
The prognosis prediction of six biomarkers in BC in Kaplan-Meier plotter database

Firstly, we analyzed the genetic alterations of biomarkers in BC by using the cBioPortal database. The results showed that 280 (12.88%) of the 2173 patients had genetic alterations. Among the six biomarkers, CACNA1H became the most commonly altered gene with a rate of 13% (Fig. 6A), and 258 (92.14%) of 280 cases had amplification that was the most frequent gene alternation in BC (Fig. 6B).

We further assessed possible significant differences in expression levels of biomarkers between tumor samples and normal samples from our TCGA dataset. The results showed that *CACNA1H*, *IL13RA1*, *NUP43*, *PGK1*, and *SDC1* were highly expressed in tumor samples, while expression of *AK3* was significantly decreased (Fig. 6C).

### Development of a nomogram to predict the prognosis of patients in breast cancer with a risk score and clinical characters

**Fig. 7:**
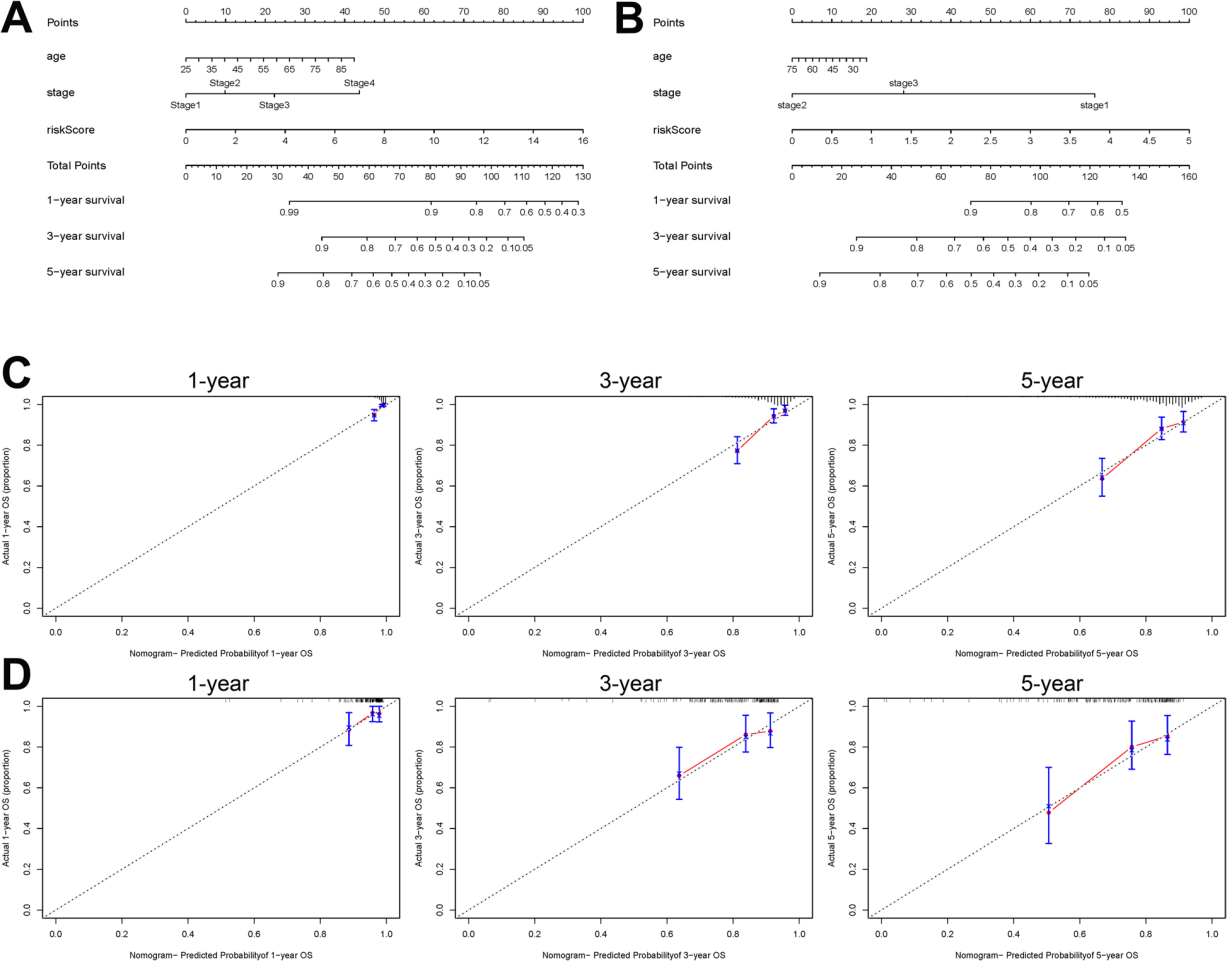
Construction of a nomogram for predicting 1-, 3-, 5-year survival. **A**, **B** The predict model was presented with a nomogram in training and validation cohorts. **C**, **D** The AUC of different indicators in nomogram in 1-, 3-, and 5-year in training and validation cohorts

To further evaluate the prognostic ability of our model and its clinical parameters, we presented the model with a nomogram both in TCGA and GEO datasets (Fig. 7A, B). Every indicator got a point, and the total number of points was calculated by the sum of all points that could predict the prognosis of patients in 1-, 3-, and 5-year survival. In training and validation cohorts, the C_index values were 0.764 and 0.677, respectively. The results of calibration curves demonstrated that the nomogram had a good accuracy of prediction on the prognosis of patients with BC (Fig. 7C, D).

### Correlation between the risk score and clinical characters

**Fig. 8:**
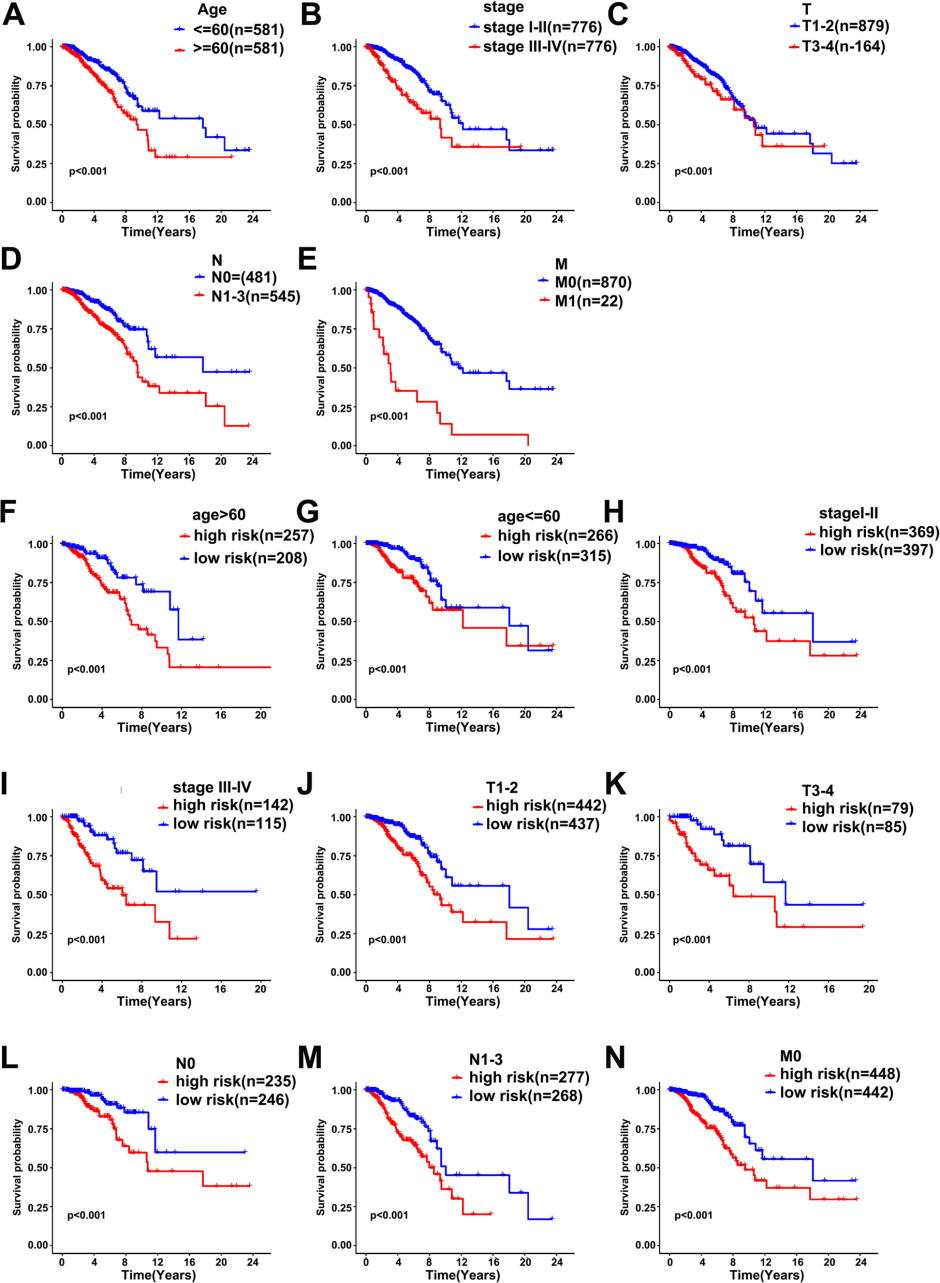
Kaplan-Meier survival analysis for BC patients with diverse clinical characters of (**A**) age, (**B**) AJCC Stage, (**C**) T stage, (**D**) N stage, (**E**) M stage. The outcomes of patients with different risk scores in subgroups of (**F**) age> 60, (**G**) age ≤ 60, (**H**) AJCC stage I‑II, (**I**) AJCC stage III‑IV, (**J**) T stage 1‑2, (**K**) T stage 3‑4, (**L**) N stage 0, (**M**) N stage 1‑3, (**N**) M stage 0

Firstly, we stratified the patients into the subgroups of age (≥ 60 and < 60), AJCC stage (I + II and III + IV), T stage (T1‑2 and T3‑4), N stage (N0 and N1‑3), and M stage (M0 and M1). The Kaplan-Meier analysis was applied by log-rank test to assess the prediction capacity of multiple clinical characters on patients with BC. The results showed that the performances of these characters were well satisfied (Fig. 8A‑E). Furthermore, we further found that the prognosis of high-risk patients in these subgroups was poorer than that of low-risk patients (Fig. 8F‑N).

### The protein expression levels of biomarkers in breast cancer tissues

**Fig. 9:**
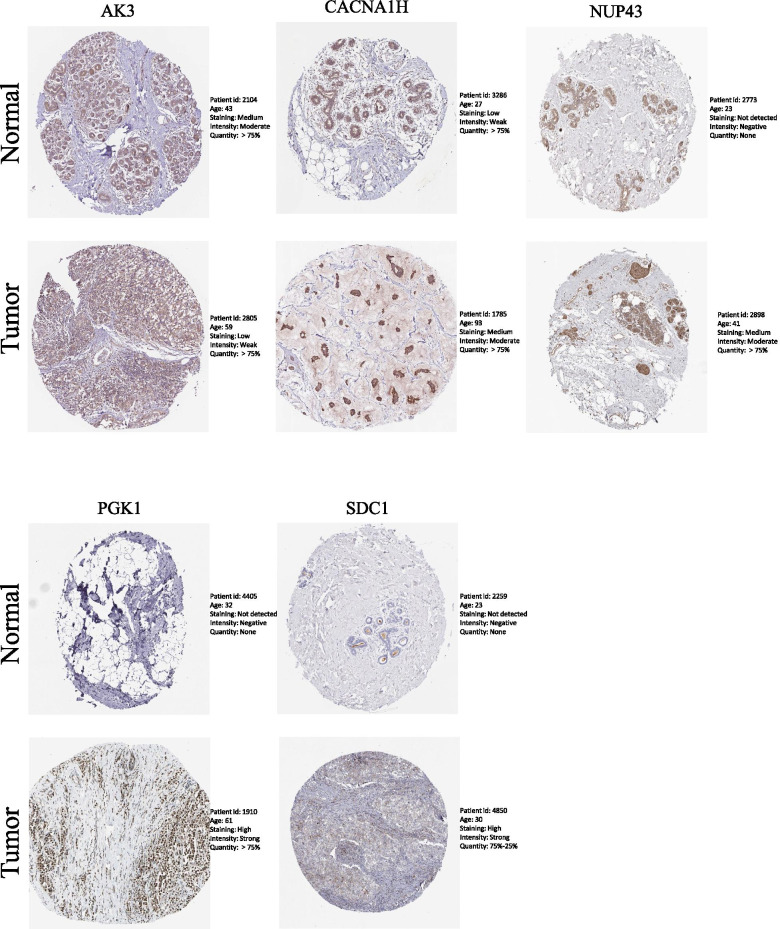
The protein expression levels of six biomarkers in breast tumoral and normal tissues from HPA

To validate the significant differences in protein expression levels of biomarkers between BC tissues and normal tissues, we performed an HPA database to assess the IHC images. Consistently, the results demonstrated that the protein expression levels of *CACNA1H*, *NUP43*, *PGK1*, and *SDC1* were higher in BC tissues compare with normal tissues, while the expression level of *AK3* was comparatively lower (Fig. 9). Unfortunately, the data of IL13RA1 was not available in the HPA database.

## Discussion

The prediction on prognosis in BC is far from satisfactory, due to the complication of its phenotypes and molecular mechanisms. Previous studies revealed that clinicopathological characters, including age, gender, and metastatic diagnosis are insufficient to accurately assess the outcomes of patients with cancer [27]. Therefore, with the development of bioinformatics, an increasing number of novel biomarkers have been constructed to get involved in the diagnosis and prediction of cancer [28, 29]. These signatures are associated with metabolism [30], immune [31], and DNA methylation [32]. The standards to judge the model are convenient, credible, and precise.

Tumor cells have the ability of unlimited proliferation and distal metastasis, and this process needs cellular metabolism to meet the requirement of adequate energy [33]. ATP and nutrients are provided by aerobic glycolysis in tumor cells that form an acidic microenvironment to promote the mutation and invasion of tumor cells [34]. Recent studies have made efforts to clarify the role of glycolysis-related genes in tumor. Ang Li et al. [27] developed a glycolysis-related signature for predicting survival of pancreatic adenocarcinoma (PAAD) that provided a novel therapy target for PAAD. Lei Zhang et al. [35] also found another similar signature to predict survival in patients with lung adenocarcinoma. Yuchao Liu et al. [36] explored the role of glycolysis in HNSCC and found that high expression levels of identified glycolysis-related genes are correlated to poor prognosis in patients with HNSCC. The abovementioned studies emphasized the importance of multiple genes rather than a single gene, which might be interfered with many factors and provide worse predictive effects. Therefore, we established a signature to predict the prognosis of survival in patients with BC, consisting of six genes.

In our study, firstly, we downloaded microarray data and clinical information from TCGA and GEO datasets, where the former was used as a training cohort, while the latter was defined as a validation cohort. Then, GSEA was applied to identify mRNAs associated with glycolysis that further found differentially expressed glycolysis-related genes. A signature was constructed through univariate and multivariate Cox regression that contained six glycolysis-related genes (*AK3*, *CACNA1H*, *IL13RA1*, *NUP43*, *PGK1*, and *SDC1*) and that would be used in the following analysis. We divided the training cohort into two groups based on the expression levels of these six biomarkers. Survival analysis showed a significant difference between high- and low-risk groups. The AUC value of the model was greater than 0.7 in both training and validation cohorts. Additionally, this risk score, age, and the stage could act as an independent prognostic index. We further developed a nomogram to predict the prognosis of patients with BC, and the results validated that our model can be used as a prognostic indicator with a good performance. The consistent results were obtained in the validation cohort, demonstrating that the GRGs model had high accuracy and could predict the prognosis of patients with BC. We further explored whether this risk score could stratify clinically defined groups of patients into subgroups of age > 60, age ≤ 60, T1‑2, T3‑4, N0, T1‑3, M0, M1, stage I‑II, and stage III‑IV. The results showed that all subgroups could be efficiently predicted by the risk score except the M1 subgroup, due to limited samples. However, when compared with traditional clinical features, the prediction model in this study had similar, even better clinical application capacity. Furthermore, the HPA database was used to explore any existence of statistical significance in the expression of six biomarkers between BC and normal tissues. We found that four of six biomarkers were highly expressed, whereas *AK3* was significantly decreased in BC tissues. The results stated above suggested that our prediction model could have better reliability and accuracy, providing a possibility to be a prospective prognostic indicator for patients with BC.

To make sense of this signature, we analyzed each biomarker. *AK3* regulates the homeostasis of adenine nucleotide composition and has been proved to have the ability of anti-tumor [37]. *AK3* could promote the BC cell migration, and its decreased expression level was found to be related to a worse prognosis in patients with BC [38]. Consistently, the expression of *AK3* was also significantly downregulated in our study. A novel glycolysis-related gene signature, including *CACNA1H*, might provide a new indicator of prediction on patients with invasive BC [24]. Another study showed that *CACNA1H* was highly expressed in luminal A and B subtypes, with the basal subtype expressing significantly lower levels of *CACNA1H* [39]. IL-13 modulates the proliferation and status of lymphocytes and has been explored in tumor research [40]. As a subtype of IL-13, high *IL13RA1* expression has been found to have a poor prognosis in patients with invasive BC and was associated with HER2- and a high Ki-67 index, showing its role of potential prognostic marker in BC [41]. In luminal A and HER2+ BC, the expression level of *NUP43* was increased and it further predicted poor prognosis [42]. A previous study demonstrated that *NUP43* plays the role of promoter in the progression of tumors [43]. Yan Zhang et al. developed glycolysis-related genes to predict the outcomes of patients in clear cell renal cell carcinoma (ccRCC); *PGK1* is one of these genes, whose high expression predicted poor prognosis in patients with ccRCC [44]. *PGK1* expression was also significantly increased in BC tissues and associated with HER2+ and positive status of ER [45]. Syndecan-1 (SDC), a heparin sulfate proteoglycan, has been found to correlate with tumor progressions [38]. Additionally, another study revealed that high *SDC1* expression is associated with increased risked of age and HER2 in patients with BC [46]. However, more investigations should be done to clarify the biological functions of these biomarkers and their interactions in BC.

Moreover, we compared the performance of our signature with others. The AUC value is an indicator to evaluate the prognostic accuracy, which means the larger the AUC value, the better the predictive ability of the biomarkers. The AUC values in this study were 0.719 and 0.702 that are higher than that in a six-gene signature associated with tumor mutation burden [47], a prognostic signature based on eight DNA repair-related genes [48], and a seven RNA signature [49]. This demonstrated that our glycolysis-related gene signature has a better performance on prediction.

## Conclusions

We constructed a glycolysis-related gene signature with *AK3*, *CACNA1H*, *IL13RA1*, *NUP43*, *PGK1*, and *SDC1* and identified them as potential prognostic biomarkers. The expression levels of these biomarkers have significant differences between BC and normal samples. Our signature can be used to predict the prognosis of patients with BC conveniently and precisely. However, there are some limitations in this study: (1) More datasets should be taken into consideration to guarantee the accuracy of results; (2) the mechanism of the six predicted genes should be explored profoundly; (3) experiments are needed to validate the results obtained from the bioinformatics analysis.

## Data Availability

All the data referred to this study are available from TCGA and GEO databases. TCGA: https://portal.gdc.cancer.gov/; GSE25065: https://www.ncbi.nlm.nih.gov/geo/query/acc.cgi?acc=GSE25065.

## Abbreviations

BC: Breast cancer
ROC: Receiver operating characteristic
HPA: Human Protein Atlas
AUC: Area under the curve
ATP: Adenosine triphosphate
HK: Hexokinase
PFK: Phosphofructokinase
PK: Pyruvate kinase
ADP: Adenosine diphosphate
PFKFB: 6-Phosphofructo-2-kinase/fructose-2,6-bisphosphatase
OS: Overall survival
PFS: Progression free survival
GLUT: Glucose transporter protein
HNSCC: Head and neck squamous cell carcinoma
GRGs: Glycolysis-related genes
GDC: Genomic Data Commons
GEO: Gene Expression Omnibus
NCBI: National Center of Biotechnology Information
AJCC: American Joint Committee on Cancer
GSEA: Gene set enrichment analysis
NES: Normalized enrichment score
IHC: Immunohistochemical
HR: Hazard ratio
PAAD: Pancreatic adenocarcinoma
